# Carpal tunnel syndrome secondary to tumoral calcinosis: a case report and review of the literature

**DOI:** 10.1186/s12891-022-05934-1

**Published:** 2022-11-08

**Authors:** Michael Abdallah, Elie Bou Sanayeh, Rami Haroun, Maria El Khoury, Majd El Hajj Moussa, Fadi Hoyek

**Affiliations:** 1 Department of Orthopedic Surgery, Notre Dame des Secours University Hospital, Jbeil, Lebanon; 2 Department of Internal Medicine, American University of Beirut Medical Center, Beirut, Lebanon; 3 Department of Diagnostic Radiology, Notre Dame des Secours University Hospital, Jbeil, Lebanon

**Keywords:** Carpal tunnel syndrome, Carpal tunnel release, Tumoral calcinosis, Wrist pain, Case report

## Abstract

**Background:**

Carpal Tunnel Syndrome (CTS) is the most prevalent peripheral nerve entrapment disease. Its pathophysiology is multifactorial and defined as idiopathic in most cases. We present a rare case of CTS secondary to tumoral calcinosis and then searched the English literature to present the details of all published cases with this entity.

Case presentation.

A 52-year-old woman presented for a one-year history of numbness and paresthesia in her right hand. The patient’s signs, symptoms, physical examination, and nerve electrodiagnostic testing suggested median nerve compression at the level of the carpal tunnel. However, a confirmatory magnetic resonance imaging of the wrist showed a localized calcareous lesion in the carpal tunnel. Subsequently, carpal tunnel release and mass excision were successfully performed with no recurrence at a 3-month interval.

**Conclusion:**

CTS secondary to tumoral calcinosis is a rare benign condition. Physicians should remain vigilant and include it in their differential diagnosis when facing a previously healthy patient presenting for chronic CTS symptoms.

## Background

Carpal tunnel syndrome (CTS), causing median nerve mononeuropathy, is the most prevalent peripheral nerve entrapment disease [1, 2]. It affects the median nerve at the level of the wrist as it crosses through the carpal tunnel [1], causing pain and numbness at the level of the volar surface of the first three digits and the radial half of the fourth digit[1]. Severe untreated cases may progressively develop weakness of the muscles innervated by the median nerve resulting in hand weakness [1]. The pathophysiology of CTS is multifactorial [3], with most cases being idiopathic due to nerve entrapment by the transverse carpal ligament. However, endocrinopathies, traumas, pregnancies, amyloidosis, or space occupying lesions (such as tumoral calcinosis, lipomas, etc.) may also contribute to secondary CTS [1, 3, 4].

Tumoral calcinosis is a unique histopathological syndrome that causes a rare benign tumor. It consists of the peri-articular deposition of a solitary, dense, calcified mass composed of calcium pyrophosphate dihydrate (CPPD) and calcium carbonate [2, 5]. It predominantly affects the elbows, shoulders, and hips, but rarely the hands [2]. In this report we present the case of a middle-aged previously healthy women that developed chronic CTS secondary to a localized compression by an idiopathic tumoral calcinosis.

## Case presentation

A 52-year-old, right-handed, previously healthy woman presented to our hospital for unprovoked numbness and worsening impairment of sensibility at the level of her right thumb, index, middle finger, and radial half of her ring finger. She also complained of impairment in everyday activities due to worsening weakness of her opponens pollicis muscle. Only slight improvement in her symptoms was reported during the past 12 months despite splinting the hand using a wrist brace, undergoing physiotherapy, and taking high doses of non-steroidal anti-inflammatory drugs (NSAIDs) and Gabapentin. The patient reported a chronic history of repetitive movements of the fingers and wrists with chronic pressure points on the right wrist.

**Fig. 1:**
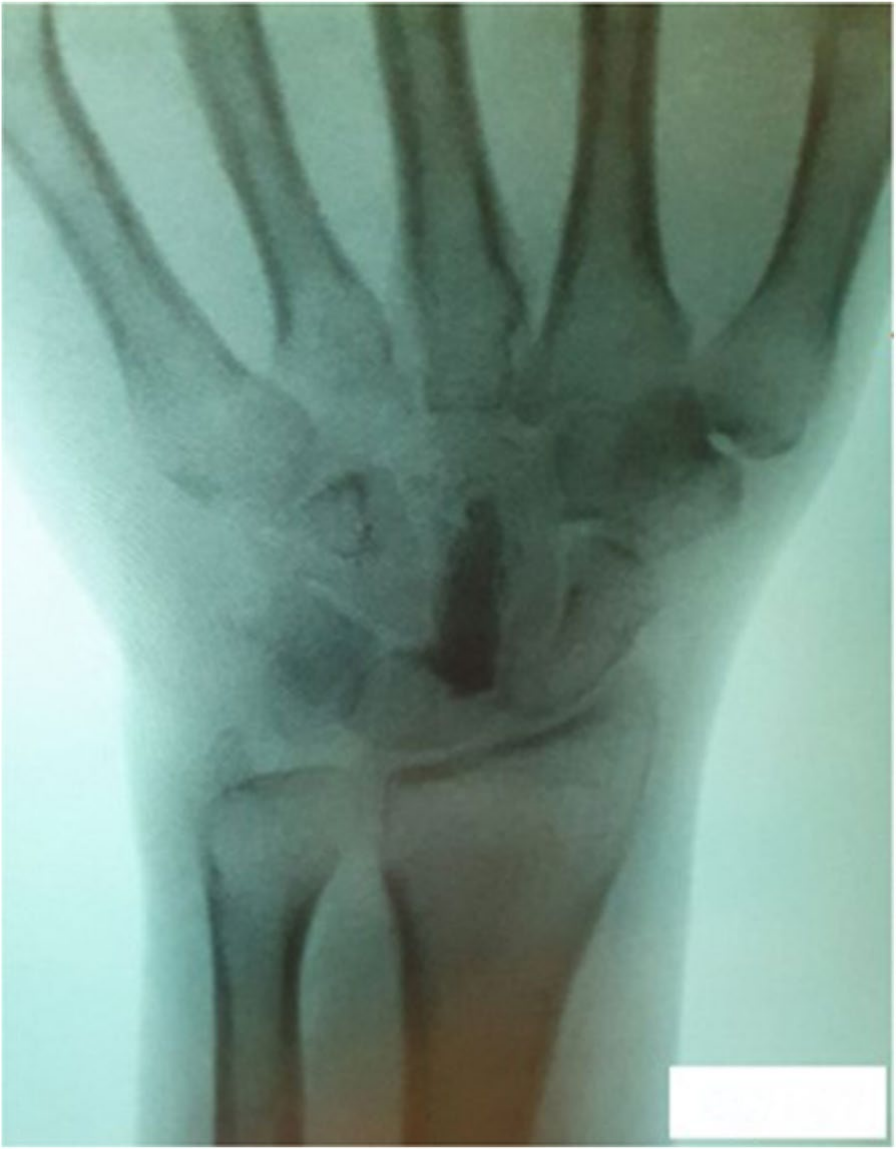
Preoperative plain radiograph showing an oval radio-opacity facing the carpal bones

On examination, the affected hand revealed no swelling or local heat. Atrophy of the thenar muscle and hypoesthesia in the distribution of the median nerve were noted. Both Phalen’s test and Tinel’s sign were positive on the right side with no restriction in the range of motion of wrist and fingers. Nerve electrodiagnostic testing suggested right median nerve compression at the level of the right carpal tunnel. An initial plain radiograph of the right wrist showed an oval radio-opacity on the volar side of the wrist joint facing the carpal bones (Fig. 1).

**Fig. 2:**
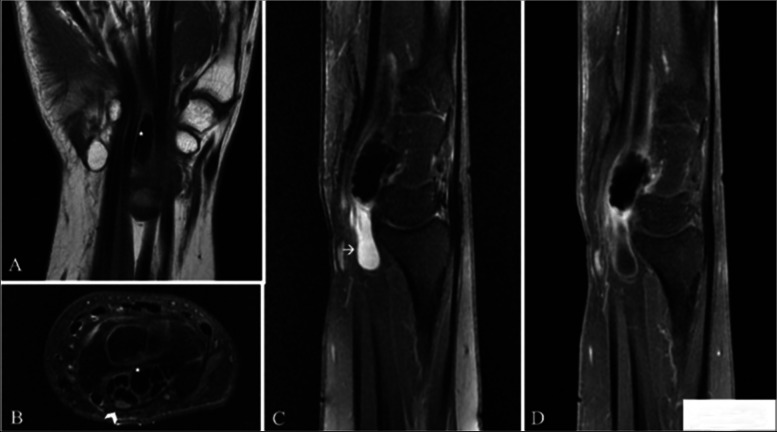
Magnetic resonance imaging of the patient’s right wrist, showing a solitary oval calcification (*), located in the carpal tunnel centrally between the flexor tendons of the wrist, at the lunatum-capitatum junction, without surrounding adherence, having low signal intensity, showing no enhancement. It is surrounded by a reactive fluid collection (**arrow**), and subtle tenosynovitis of the flexor’s tendon sheaths, with mild compression of the median nerve (**arrowhead**). **A** Coronal T1-weighted Fast Spin Echo sequence. **B** Axial and **C** Sagittal Proton Density (PD) fat saturated sequences. **D** Post contrast administration sagittal T1-weighted fat saturated sequence

A confirmatory magnetic resonance imaging (MRI) of the right hand and wrist showed a solitary oval calcification (low-intensity lesion both in T1WI and T2WI) measuring 2 × 0.8 × 0.6 cm (cm), located in the carpal tunnel centrally between the flexor tendons of the wrist, at the lunatum-capitatum junction, without surrounding adherence (the boundary between the lesion and the surrounding tissues was clear) (Fig. 2A and B). The lesion is also surrounded by a reactive fluid collection (Fig. 2C). The MRI also showed subtle tenosynovitis of the flexor’s tendon sheaths, with mild compression of the median nerve (Fig. 2B). The patient’s full blood count, vitamin D, calcium, phosphate, electrolytes, uric acid, urea, creatinine, and alkaline phosphatase were within normal range. Other laboratory data including an endocrine and rheumatology panel were also normal. Subsequently, the patient was diagnosed with CTS secondary to a localized calcareous mass.

**Fig. 3:**
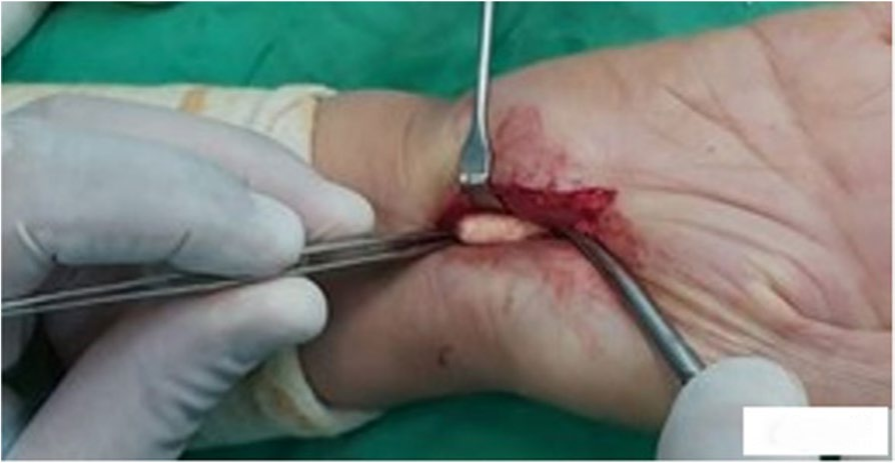
Incision performed on the volar side of the right wrist

**Fig. 4:**
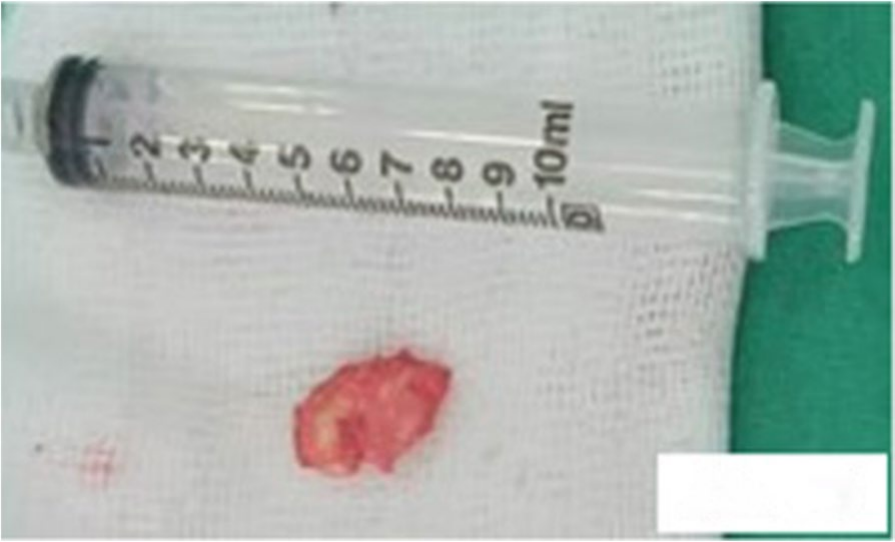
The removed 2.1 by 1.0 cm mass

Given that conservative treatment was ineffective, the patient’s condition was managed by open incisional carpal tunnel release. An incision of approximately 4 cm was performed on the volar side of the right wrist facing the third metacarpal bone (Fig. 3). The palmar aponeurosis was then dissected, and the flexor retinaculum was located and transected. The white calcareous tumor was lying over the carpal bones of the osteofibrous canal, and it was only visualized after retracting the median nerve. A 2.1 by 1.0 cm mass (Fig. 4) was easily removed with no adhesion to surrounding tissues. Histological sections showed calcified deposits encased in a fibrocartilaginous tissue with inflammatory infiltrates composed of giant cell granulomas. These findings supported the diagnosis of tumoral calcinosis [6, 7].

**Fig. 5:**
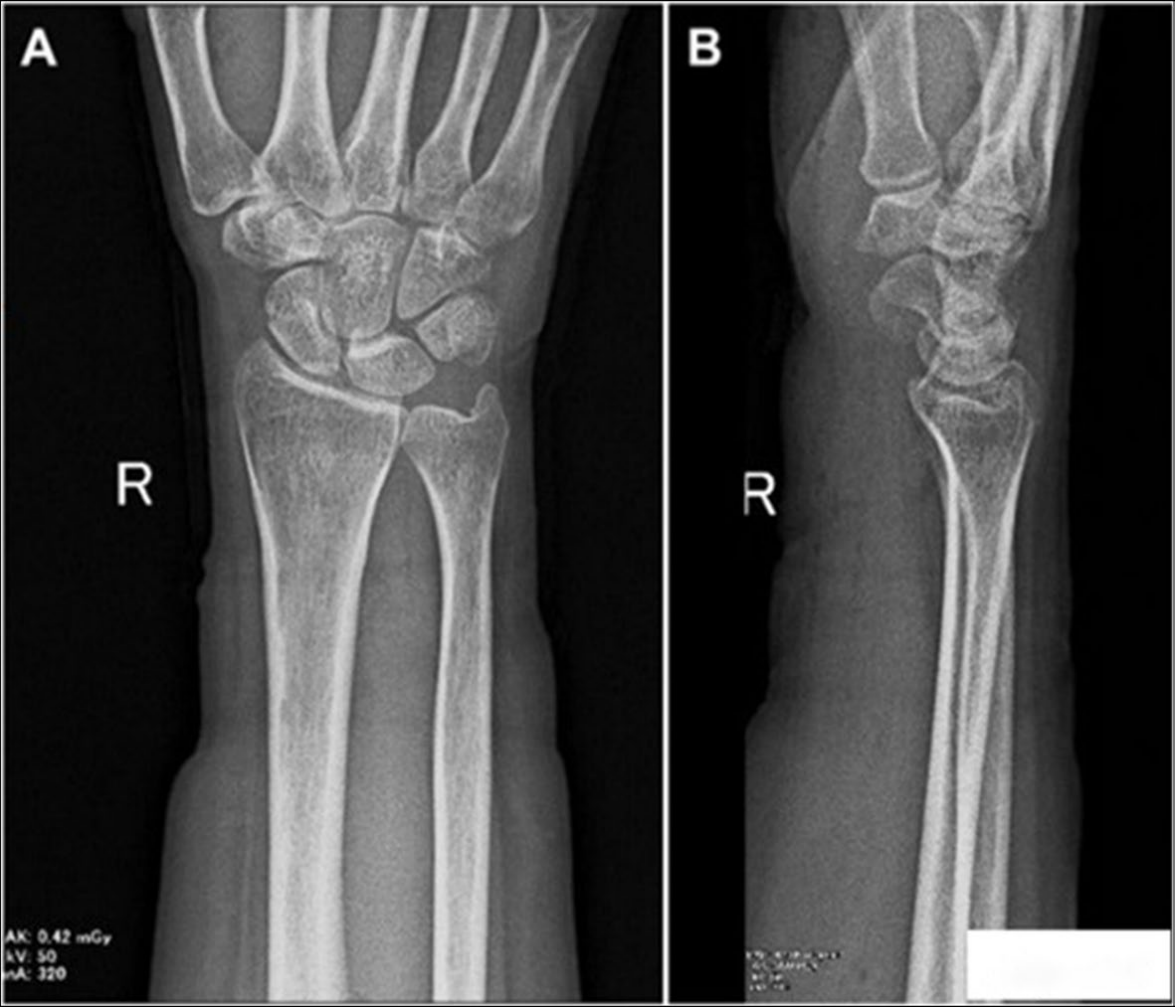
Postoperative plain radiograph following removal of the calcification. **A** Postero-anterior view. **B** Lateral view

During her follow up, three months following the surgery, no clinical or radio-graphical signs (Fig. 5) of recurrence were noted and the patient reported complete resolution of her symptoms.

## Literature review

Research for available data since 1980 was conducted in PubMed database using the option “Advanced Search” and selecting “Title” in the search builder and the following combinations in the search box: “tumoral calcinosis”, “calcified mass”, “calcium deposition”, “calcification”, “carpal tunnel”, “carpal tunnel syndrome”, “peri-articular calcification”, and “median nerve”. Available data as abstracts or full text articles and related citations and references were reviewed, and in selected cases, full text articles were purchased. Relevant information was included in Table 1.

**Table 1:** Cases of carpal tunnel syndrome secondary to tumoral calcinosis published in the English literature

First author’s name	Year of publication	Age of patient (in years), gender	Past medical history	Diagnostic modality	Size of the tumor in cm	Treatment modality	Histopathological findings	Suggested etiology
Hecht et al.[10]	1980	41, F	None	X-ray of the wrist joint	Not mentioned	Surgical resection	Not mentioned	Not mentioned
De et al.[17]	1983	32, F	None	X-ray of the wrist joint	2.5 × 0.8	Surgical resection	Not mentioned	Primary calcinosis Nonfamilial
Weiber et al.[2]	1987	63, F	None	Only clinical	3 × 1	Surgical resection	Rounded psammoma-like bodies and granulation tissue containing histyocytes and osteoclast-like giant cells	Primary calcinosis Nonfamilial
Ali et al.[18]	1988	41, F	None	X-ray of the wrist joint	Not mentioned	Surgical resection	No true capsule and the mass contains calcium phosphate in a fibrous proliferation, giant cells, and lymphocytes	Primary calcinosis Nonfamilial
Bostrom et al.[19]	1993	38, F	None	X-ray of the wrist joint	Not mentioned	Surgical resection	Not mentioned	Not mentioned
Bostrom et al.[19]	1993	64, F	Hypothyroidism	X-ray of the wrist joint	0.5 × 0.9	Surgical resection	Not mentioned	Not mentioned
Knight et al.[20]	1993	50, F	None	X-ray of the wrist joint	Not mentioned	Surgical resection	Acute inflammatory changes and an area of calcification	Primary calcinosis Nonfamilial
Asami et al.[12]	1998	52, M	ESRD on HD	Electrophysiological study	Large mass (exact size not mentioned)	Surgical resection	Fibrous connective tissue+ calcified area surrounded by mononuclear cells + polynuclear cells with lipi-containing cytoplasm and foreign body giant cells	Secondary: Hyperphosphatemia
Takada et al.[8]	2000	63, F	Not mentioned	Electrophysiological study + X-ray of the wrist joint	2 × 1.2	Surgical resection	Amorphous calcified material encapsulated with fibrous membrane without inflammation	Primary calcinosis Nonfamilial
Cofan et al.[13]	2002	25, F	SLE, ESRD on HD	Electrophysiological study + CT and MRI of the wrist joint	4 × 3.4	Surgical resection + Increase in the duration of dialysis+ correction of the hyperphosphatemia + parathyroidectomy	Calcified mass surrounded by granulation tissue with histyocytes and some multinucleated cells	Secondary: Severe hypercalcemia and hyperphosphataemia from hyperparathyroididsm and excessive calcitriol administration
Sensui et al.[21]	2003	64, F	Not mentioned	Electrophysiological study + CT of the wrist joint	Not mentioned	Surgical resection	Amorphous calcification	Primary calcinosis Nonfamilial
Pai et al.[22]	2009	64, F	None	X-ray and CT of the wrist joint	2 × 2	Surgical resection	Hydroxyapatite crystals	Primary calcinosis Nonfamilial
Kang et al.[23]	2009	55, F	None	CT and MRI of the wrist joint	Not mentioned	Surgical resection	Amorphous calcified material encapsulated with fibrous membrane	Not mentioned
Kang et al.[23]	2009	78, F	None	CT and MRI of the wrist joint	Not mentioned	Surgical resection	Amorphous calcified material encapsulated with fibrous membrane	Not mentioned
Kang et al.[23]	2009	55, F	None	CT and MRI of the wrist joint	Not mentioned	Surgical resection	Amorphous calcified material encapsulated with fibrous membrane	Not mentioned
Inui et al.[4]	2015	54, F	None	Electrophysiological study + CT and MRI of the wrist joint	2.7 × 1.2	Surgical resection	Basophile deposition inside the fibrous connective tissue. Made of 82% calcium phosphate and 17% calcium carbonate	Primary calcinosis Nonfamilial
Kwon et al.[3]	2018	45, F	None	Electrophysiological study + US and MRI of the wrist joint	1.19 × 0.96	Surgical resection	Calcified nodules	Primary calcinosis Nonfamilial
Cheng et al.[24]	2019	57, F	None	X-ray and MRI of the wrist joint	1.3 × 0.8 × 1	Surgical resection	Calcified nodule	Primary calcinosis Nonfamilial
Cheng et al.[24]	2019	52, F	Type 2 diabetes mellitus	Electrophysiological study + X-ray and ultrasound of the wrist joint	0.6 × 0.6 × 1.3	Surgical resection	Calcified nodule	Primary calcinosis Nonfamilial

*CT* Computerized Tomography, *ESRD* End-Stage Renal Disease, *F* Female, *HD* Hemodialysis, *M* Male, *MRI* Magnetic Resonance Imaging, *SLE* Systemic Lupus Erythematosus, *US* Ultrasound

## Discussion and conclusion

In this manuscript, we reported a rare case of CTS secondary to an abnormal calcareous lesion within the carpal tunnel. We also searched for all similar cases in the English literature since 1980. We found a total of 19 cases from 15 articles, with patients’ mean age of 52.26 years.

CTS is the most prevalent peripheral nerve-entrapment disease. While most cases are idiopathic, some are secondary to vascular abnormalities, tenosynovitis, malunited distal radial fractures or space-occupying lesions [4]. The latter are rare causes of CTS and they include synovial sarcomas, fibromas of the tendon sheath, calcified lesions, etc [4]. Numerous conditions can trigger these depositions such as pseudogout, gout, idiopathic calcification or tumoral calcinosis [8].

CTS is suspected clinically, and electrophysiological studies would confirm and evaluate the severity of entrapment [1]. Usually, idiopathic cases will engender bilateral CTS. Consequently, unilateral CTS may warrant further examination to rule out an underlying etiology [4]. MRI or ultrasound of the wrist are useful in such cases to establish the diagnosis [1]. At last, cases in which a mass is surgically removed, histopathological examination is essential to confirm its nature and composition. In our case, the tumor was composed of crystal phosphate, confirming the diagnosis of tumoral calcinosis.

Tumoral calcinosis is a benign condition [9]. It is usually asymptomatic but can cause nerve compression in some cases [1]. When compressing the median nerve at the level of the wrist, it can promote CTS. This combination was rarely mentioned in the literature since it was first reported by Hecht et al. in 1980. [10] Many criteria were proposed to diagnose tumoral calcinosis [11], however, it is currently considered as any peri-articular calcium-deposit-like tumor regardless of the patient’s preexisting disease, age, or gender [7]. Two subtypes of tumoral calcinosis were described: primary and secondary. The secondary type is associated with other conditions such as chronic renal failure, cancer, hyperparathyroidism, hypervitaminosis D, connective tissue diseases, etc [7]. These conditions must always be ruled out by a complete laboratory workup[7]. In the literature, 17 of the reported cases were diagnosed in patients with no significant prior medical history and only two were seen in patients with end-stage renal disease [12, 13].

Regarding the primary type, which is the case in our patient, no associated diseases are thought to be involved; and it is characterized by the presence of a solitary nodule [9] that appears usually more prevalent among Africans living in tropical or subtropical regions [6, 14]. Namba et al. pointed that repetitive mechanical traumas to the carpal ligaments, such as in our patient, might be the cause of tumoral calcinosis. [5] Multiple micro-traumas lead to transient hyperphosphatemia secondary to phosphate release from injured cells into the extracellular space [15]. With time, calcium phosphorus products will accumulate and subsequently calcify [15].

Primary tumoral calcinosis can also be classified into normophosphatemic and hyperphosphatemic [7]. In normophosphatemic calcinosis, which is the case in our patient, serum concentrations of phosphate and calcium are within normal range [7]. However, patients with hyperphosphatemic calcinosis, have high serum phosphate concentrations with normal calcium concentrations, and they usually have a familial history of calcinosis secondary to abnormal phosphate resorption in the distal tubule [7]. From our review, cases in which phosphate levels were measured, none of the patients with primary calcinosis had abnormal results.

Regarding the curative therapy, CTS is initially managed conservatively (rest and lifestyle optimization) [1]. When conservative measures fail, surgical release of the transverse carpal ligament becomes indicated[1]. In patients with primary or idiopathic tumoral calcinosis causing CTS, relieving the nerve compression by removing the tumor is vital for symptoms’ relief and long-term complications’ avoidance. It is noteworthy that tumoral calcinosis progresses in three stages and the management of CTS secondary to this entity must be tailored based on its stage of development [16]. Medical treatment based on phosphate dietary depletion and aluminum hydroxide administration is superior to surgery during the first two stages where the aggregation of foamy macrophages followed by calcification and formation of a poorly localized mass occur [16]; while surgical resection is the definite treatment in the quiescent third stage in which the mass is totally calcified [7, 16]. In regard to the secondary type, medical therapy must be adopted first to control the underlying causative disease prior to any surgical attempt [9]. In this category, most cases are seen in patients maintained on hemodialysis for end-stage renal disease. For the two similar cases reported in the literature, the calcification was attributed to the rise in calcium-phosphorus products secondary to hyperparathyroidism, excess supplementation in calcitriol or calcium carbonate, suboptimal phosphorus-chelating therapy, and inadequate dialysis [12, 13]. Subsequently, prior to surgically removing the tumor, it is necessary to address these factors first by extending the dialysis time, treating hyperphosphatemia, and optimizing the calcitriol dosages. Finally, in indicated cases, mass reduction was reported following the creation of a negative calcium balance through renal transplantation or parathyroidectomy [12, 13]. In our review, all cases of primary calcinosis were treated surgically, while those with the secondary type received, in addition to the surgical excision, optimization of their renal medical management. All 19 reported cases had complete resolution of their symptoms directly after the surgical intervention and no cases of recurrence were reported.

This case advances our knowledge concerning the clinical presentation and management of CTS secondary to tumoral calcinosis. Tumoral calcinosis is an uncommon and underdiagnosed entity in patients presenting with symptoms of chronic CTS. The diagnosis might be challenging but signs are often present on plain radiograph of the wrist and MRI can confirm the diagnosis. Physicians should remain vigilant and include tumoral calcinosis in their differential diagnosis given that carpal tunnel release with mass excision provides excellent results.

## Data Availability

Data sharing is not applicable to this article as no datasets were generated or analyzed during the current study.

## Abbreviations

CTS: Carpal Tunnel Syndrome
CPPD: Calcium Pyrophosphate Dihydrate
NSAIDs: Non-Steroidal Anti-Inflammatory Drugs
MRI: Magnetic Resonance Imaging
cm: Centimeters
CT: Computerized Tomography
ESRD: End-Stage Renal Disease
F: Female
HD: Hemodialysis
M: Male
SLE: Systemic Lupus Erythematosus
US: Ultrasound
